# Supplementary material for: Origin of the low critical observing temperature of the quantum anomalous Hall effect in V-doped (Bi, Sb)2Te3 film
Source: Sci Rep. 2016 Sep 7;6:32732. doi: 10.1038/srep32732 (PMC5013448; doi:10.1038/srep32732)
Supplement: Supplementary Information [file srep32732-s1.pdf]

Origin of the low critical observing temperature of the quantum anomalous Hall effect in V-doped $(Bi, Sb)_2Te_3$ film

W. Li[1**], M. Claassen[1**], Cui-Zu Chang[2**], B. Moritz[1], T. Jia[1, 3], C. Zhang[1], S. Rebec[1, 3], J. J. Lee[1, 3], M. Hashimoto[4], D.-H. Lu[4], R. G. Moore[1], J. S. Moodera[2, 5], T.P. Devereaux[1, 3*], & Z.-X. Shen[1, 3*]

[1]*Stanford Institute for Materials and Energy Sciences, SLAC National Accelerator Laboratory and Stanford University, Menlo Park, California 94025, USA*

[2]*Francis Bitter Magnet Lab, Massachusetts Institute of Technology, Cambridge, MA 02139, USA*

[3]*Departments of Physics and Applied Physics, and Geballe Laboratory for Advanced Materials, Stanford University, Stanford, California 94305, USA*

[4]*Stanford Synchrotron Radiation Lightsource, SLAC National Accelerator Laboratory, Menlo Park, California 94025, USA*

[5]*Department of Physics, Massachusetts Institute of Technology, Cambridge, MA 02139, USA*

**To whom correspondence should be addressed: zxshen@stanford.edu, tpd@stanford.edu*

*** These authors contribute equally to this work*

Content

Supplementary Note 1: Location of the Dirac point.

Supplementary Note 2: Decapping procesure of the QAH films.

Supplementary Figure S4. Band dispersions along cut 1 direction in Fig. 3.

Supplementary Figure S5. Contrasting the effects of exchange coupling and internal magnetic field.

Supplementary Note 1: Location of the Dirac point

As shown in Fig. S1, the bands of the QAH sample mostly reveal the Dirac cone structure below the Dirac point. Usually, in the (Bi, Sb)$_2$Te$_3$ topological insulator family, one could find that the lower part of surface bands is always much broader than the upper ones. Another issue may also cause bands broadening is the V-doping in (Bi, Sb)$_2$Te$_3$. Those two points together make the determination of the energy of the Dirac point (E_D) more difficult. To decrease the uncertainty, two methods were carried out to determine the location of the Dirac point.

As shown in Fig. S1, direct linear fitting of the surface bands along two different high symmetric directions give the E_D ~ -59 meV.

Figure S2a shows the momentum distribution curves (MDC) along the Γ-M direction. The two MDC peaks of the surface bands could not be distinguished and merge into a single peak from energy range of E_F to -110 meV, which could be also clearly observed in the corresponding second derivative with respect to momentum in Fig. S2b. The single MDC peaks were fitted with Lorentzian profiles. Comparison of full width at half maximum (FWHM) of the single MDC peaks among this energy range give rise to the E_D ~ -54 meV (the energy of which has the sharpest MDC peak). This is comparable with the value determined by direct linear fitting.

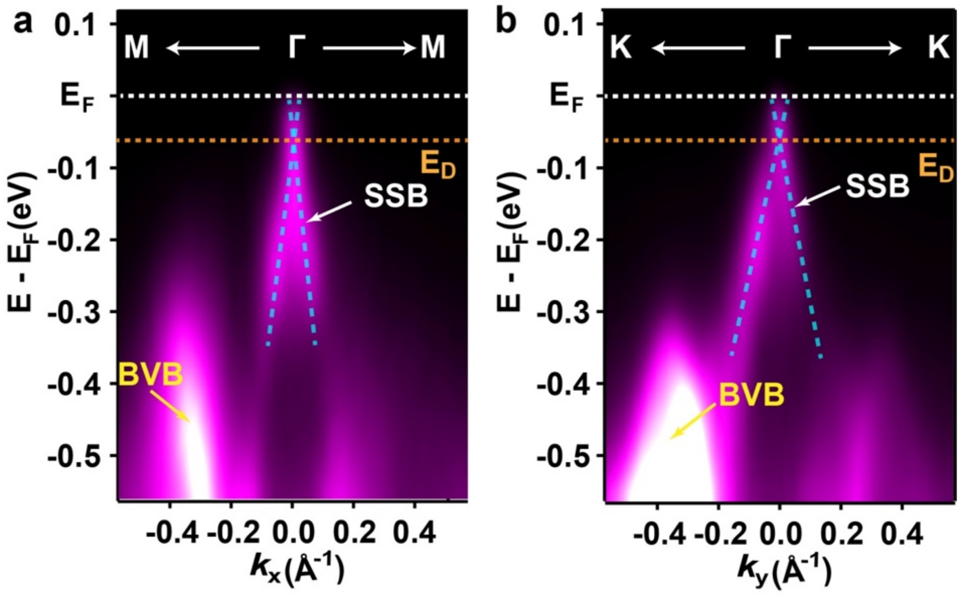

Figure S1 | Band dispersions of a QAH sample and the determination of the E_D. The VBM along Γ-M direction is closer to the DP than that along the Γ-K direction.

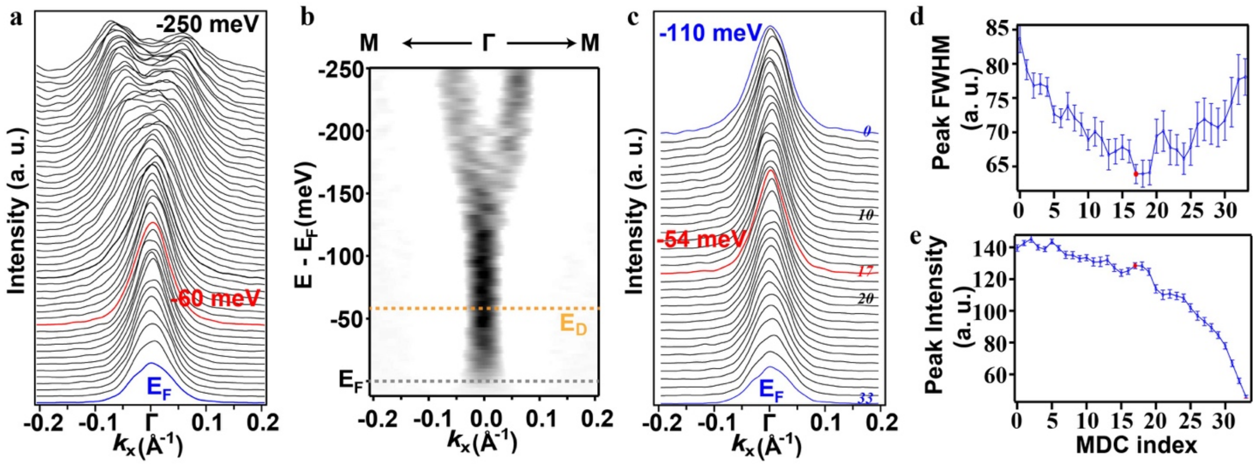

Figure S2 | Determination of the E_D by comparison of the FWHM of MDC peaks. MDCs **a** near the Dirac point (the highlight MDC is with the energy close to the E_D obtained by direct linear fitting) and the corresponding second derivative with respect to momentum **b**. **c**, the MDCs in the energy region, where the double peaks of the surface bands merge into one single peak. **d** and **e**, peak FWHM and intensity of the MDCs in **c**. The sharpest peak arises from the MDC with the energy of –54 meV.

Supplementary Note 2: Decapping procesure of the QAH films

To remove the Te capping layer of QAH films in UHV chamber before ARPES experiment, very careful annealing of the sample is performed.

Since the QAH film starts to decompose at 300 °C, and Te capping layer could be removed between the temperature range of 200 -280 °C, it is good to perform the de-capping procedure at lower temperature, which is challenging for typical infrared radiation (IR) temperature measurement. Therefore, we gradually increased the annealing current and simultaneously monitored the samples by RHEED. Appearance of the sharp RHEED pattern of the QAH films (Fig. S3a) indicates the proper de-capping temperature. Higher annealing temperature (higher current) would cause the decomposition of the QAH film and signature of substrate in RHEED pattern (Fig. S3b). Several rounds of the annealing procedures were performed to optimize the de-capping temperature.

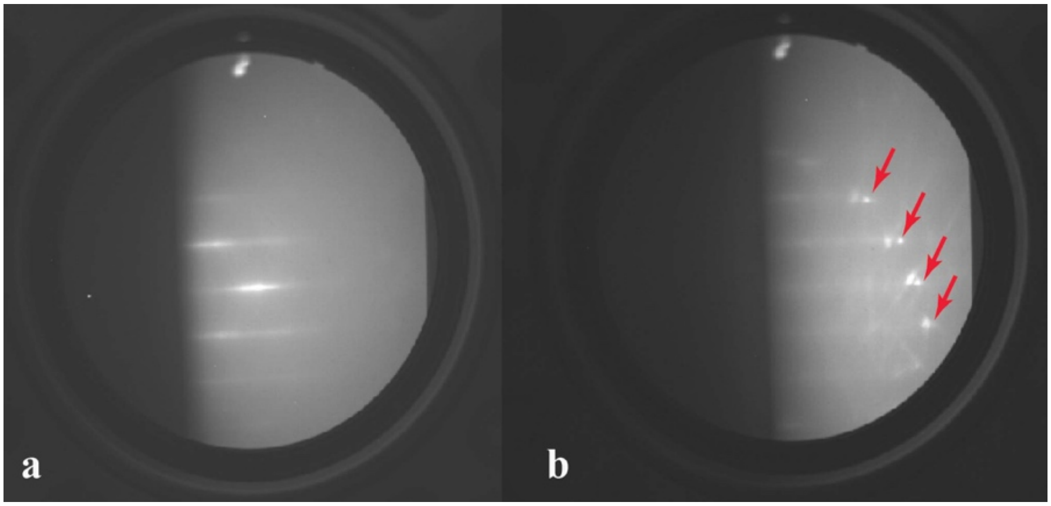

Figure S3 | RHEED patterns during de-capping procedures. **a**, sharp RHEED pattern indicating the optimal de-capping temperature. **b**, signature of the substrate marked by red arrows with higher annealing temperature.

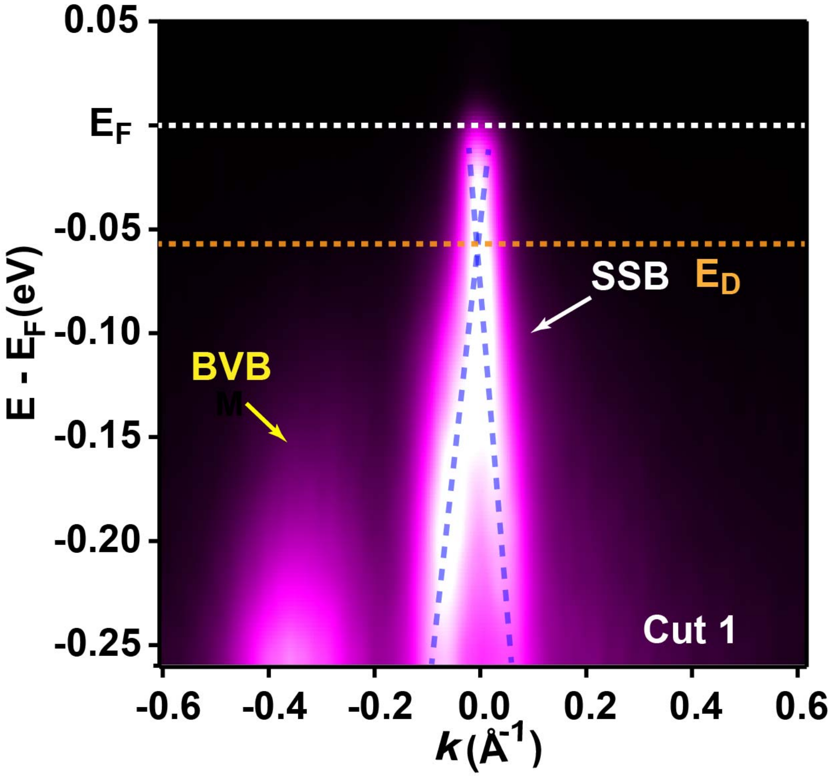

Figure S4 | Band dispersions along cut 1 direction in Fig. 3.

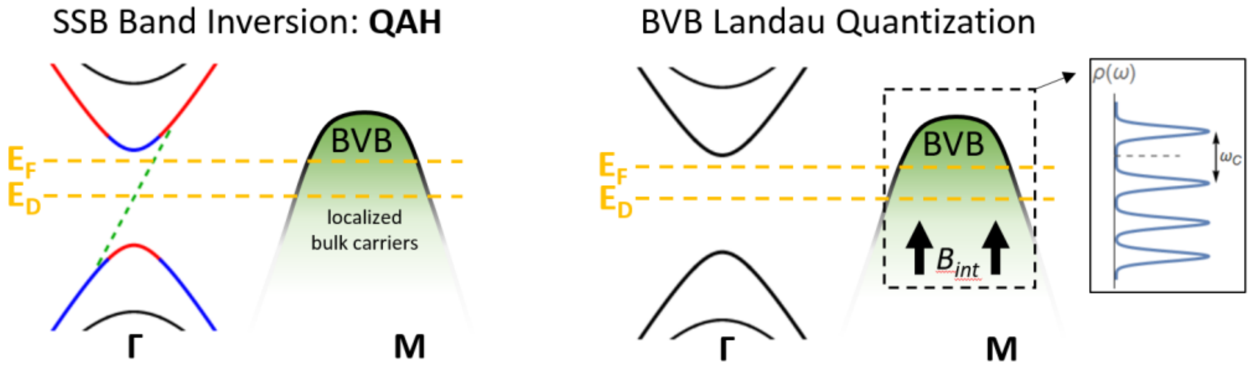

Figure S5 | Contrasting the effects of exchange coupling and internal magnetic field: (left) Exchange coupling between magnetic moments and hybridized top and bottom surface Dirac cones inverts the Dirac mass for one spin orientation only, inducing a chiral state at the thin film edge. Anderson localization ensures an insulating 2D bulk at low temperatures. (right) Internal magnetic field of V moments quantizes electron motion in the BVB, surpassing the localization energy. Landau quantization of the BVB analogously induces a chiral edge state.
